# Prenatal quantification of human foetal lung and liver elasticities between 24 and 39 weeks of gestation using 2D shear wave elastography

**DOI:** 10.1007/s00330-022-08654-1

**Published:** 2022-03-10

**Authors:** Camille Nallet, Lionel Pazart, Claire Cochet, Chrystelle Vidal, Jean-Patrick Metz, Emmanuelle Jacquet, Guillaume Gorincour, Nicolas Mottet

**Affiliations:** 1 Pôle Mère-Femme, Department of Obstetrics and Gynecology, University Hospital of Besancon, University of Franche-Comte, Boulevard Alexandre Fleming, 25000 Besançon, France; 2 Centre d’investigation Clinique-Innovation Technologique 1431, INSERM, University Hospital of Besançon, 25000 Besançon, France; 3 Department of Applied Mechanics, Université de Bourgogne Franche-Comté, FEMTO-ST Institute, UFC/CNRS/ENSMM/UTBM, 25000 Besançon, France; 4 Institut Méditerranéen d’Imagerie Médicale Appliquée à la Gynécologie, la Grossesse et l’Enfance (IMAGE 2), 6 Rue Rocca, 13008 Marseille, France; 5 Nanomedicine Lab, Imagery and Therapeutics, EA4662, University of Franche-Comte, 25000 Besancon, France

**Keywords:** Foetal lung, Foetal liver, Shear wave elastography, Elasticity, Dispersion

## Abstract

**Objectives:**

To quantify and model normal foetal lung and liver elasticities between 24 and 39 weeks of gestation (WG) using two-dimensional shear wave elastography (2D-SWE). To assess the impact of the distance between the probe and the target organ on the estimation of elasticity values.

**Methods:**

Measurements of normal foetal lungs and liver elasticity were prospectively repeated monthly between 24 and 39 WG in 72 foetuses using 2D-SWE. Elasticity was quantified in the proximal lung and in the region inside the hepatic portal sinus. The distance between the probe and the target organ was recorded. Trajectories representing foetal lung and liver maturation from at least 3 measurements over time were modelled.

**Results:**

The average elasticity for the lung and liver was significantly different from 24 WG to 36 WG (*p <* 0.01). Liver elasticity increased during gestation (3.86 kPa at 24 WG versus 4.45 kPa at 39 WG). From 24 WG to 32 WG, lung elasticity gradually increased (4.12kPa at 24 WG, 4.91kPa at 28 WG, 5.03kPa at 32 WG, *p* < 0.002). After 32 WG, lung elasticity decreased to 4.54kPa at 36 WG and 3.94kPa at 39 WG. The dispersion of the average elasticity values was greater for the lung than for the liver (*p* < 0.0001). Variation in the elasticity values was less important for the liver than for the lung. The values were considered valid and repeatable except for a probe-lung distance above 8cm.

**Conclusion:**

Foetal lung and liver elasticities evolve differently through gestation. This could reflect the tissue maturation of both organs during gestation.

**Trial registration:**

clinicaltrials.gov identifier: NCT03834805

**Key Points:**

*• Prenatal quantification of foetal lung elasticity using 2D shear wave elastography could be a new prenatal parameter for exploring foetal lung maturity.*

*• Liver elasticity increased progressively from 24 weeks of gestation (WG) to 39 WG, while lung elasticity increased first between 24 and 32 WG and then decreased after 32 WG.*

*• The values of elasticity are considered valid and repeatable except for a probe-lung distance above 8cm.*

## Introduction

Foetal lung development is accompanied by cell proliferation, intense renewal of the extracellular matrix and increased deposits of elastin responsible for changes in tissue biomechanical characteristics during gestation [1]. According to ex vivo experimental studies, normal lung stiffness ranges from 0.5 to 15 kPa, and the process of normal foetal lung development is dependent on mild tissue distension [2, 3]. The natural stretching of the foetal lung during gestation is important for regulating matrix cellular differentiation and controlling surfactant synthesis [3]. All these interactions and histological changes supported by the collagen and elastic fibre system influence biomechanical properties and hence the functioning of the lung. Prenatal assessment of foetal lung elasticity could be a relevant approach to studying foetal lung maturity during pregnancy.

All living tissues display viscoelastic behaviour, which is characterized by time- and frequency-dependent behaviour of the material responses [4]. In shear wave elastography (SWE), the tissue is perturbed by an acoustic radiation force to generate shear waves whose propagation speed can be linked to the elastic properties of the tissue using mathematical models [5]. Quantitative evaluation of the stiffness is expressed either in terms of shear wave speed (SWS) (m.s^-1^) or Young’s modulus (YM) (kPa) [5–7]. The study of deep organs is possible with 2D-SWE because this method does not require any compression-relaxation sequence on the target organ. Nevertheless, the distance between the probe and deep organs can limit the accurate measurement of SWS. This technique has been used in several organs (liver, breast, placenta, cervix) [7–11], and some studies have reported interesting results regarding the exploration of non-human foetal organs with 2D-SWE [12, 13].

In a preliminary pilot study (the ELASTOMAP study), we concluded that quantitative foetal lung and liver stiffness measurements during gestation were feasible using 2D-SWE with acceptable reproducibility [14]. We explored both the foetal lung and liver because the latter has been used as a reference organ to assess foetal lung maturity in several studies using ultrasound or MRI [15–17]. Considering that gestational age is one of the most important factors determining foetal lung maturation, the increased lung stiffness observed during the studied period could reflect lung development and maturity. However, in this previous prospective case-control study, foetuses were stratified according to gestational age between 24 and 34 weeks of gestation (WG), and individual evolution of foetal lung elasticity was not studied. The protocol was not designed to study the normal value of foetal lung or liver elasticity during gestation. Moreover, data concerning foetal lung elasticity after 34 WG were not available, while 39 WG is an important threshold in terms of lung maturity in current clinical practice [18, 19].

The primary objective of the current study was to quantify the elasticity evolution of normal foetal lungs and liver in foetuses between 24 and 39 WG using 2D-SWE. The secondary objective was to assess the impact of the distance between the probe and the target organ on the estimation of elasticity values for both organs.

## Methods

### Study design

A prospective observational study was performed at the University Hospital of Besançon, (France), and at IMAGE Institute (Marseille, France) from April 2019 to January 2020 in strict accordance with the ethical guidelines of the Declaration of Helsinki. The study was approved by the human ethical research committee (Comité de Protection des Personnes SUD EST VI, process number AU1437) and the *French National Agency for Medicines and Health Products Safety* (process number 2018-A01607-48). The study was registered at clinicaltrials.gov with the number NCT03834805. All the participants provided written informed consent.

### Study population

Measurements of normal foetal lungs and liver elasticities were repeated monthly for the same foetus using 2D-SWE between 24 and 39 WG. Pregnant women were included at 24 WG+/-1 week, and ultrasounds with 2D-SWE were performed at 28, 32, 36 and 39 WG+/-1 week. It was determined that 72 patients should be included in the current study (see section “Statistical analysis”). The inclusion criteria were as follows: pregnant women aged 18 years or older, singleton pregnancy, eutrophic foetus and signature of consent. Maternal exclusion criteria were BMI > 30 kg/m^2^, premature rupture of membranes, arterial hypertension, pre-eclampsia, gestational diabetes, prescription of maternal corticosteroids for foetal lung maturation and women under a legal incapacity. Foetal and neonatal exclusion criteria were as follows: foetal lung or liver pathologies, intra-uterine growth restriction (IUGR) < 10^th^ percentile, birth weight < 10^th^ percentile, respiratory distress syndrome (RDS) or transient tachypnoea with a Silverman score > 4 and postnatal diagnosis of structural or chromosomal abnormalities. All these conditions could influence normal lung maturity. Moreover, we decided to exclude foetal weight estimation or birthweight < 10^th^ percentile because Alison et al found that postnatal liver stiffness was significantly higher for foetuses with IUGR [8].

### Prenatal variables and measuring technique

**Fig. 1:**
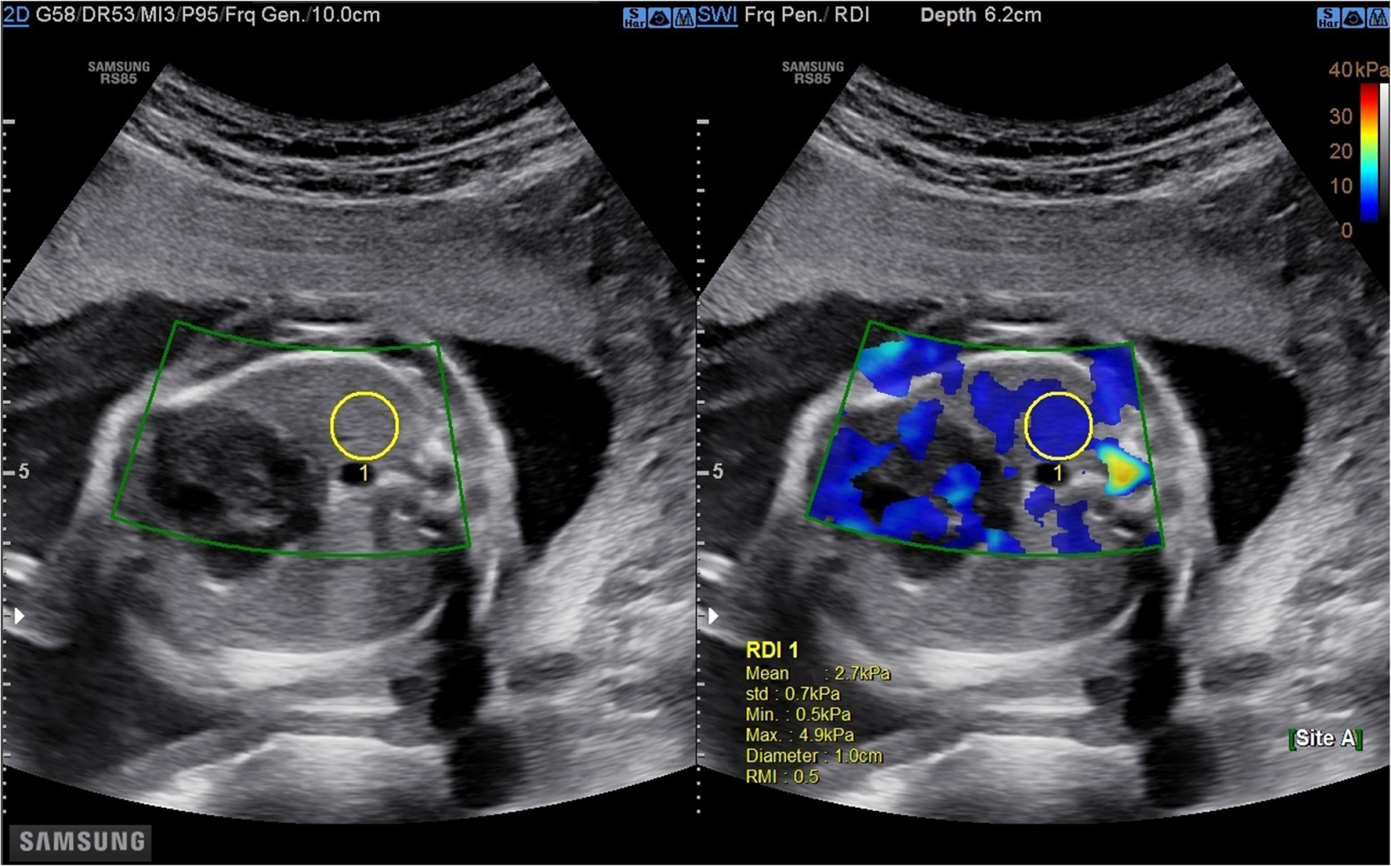
Elasticity measurement of the proximal foetal lung with 2D-SWE in the region behind the plane passing through the atria after obtaining a B-mode image of a four-chamber view of the foetal heart. The colour box represents the elastogram (blue), and the yellow circle represents the region of interest (1 cm) where the elasticity measurement is acquired. The RMI (Reliability Measurement Index) value (0.5) is expressed below the obtained mean elasticity measurement of 2.7 kPa

**Fig. 2:**
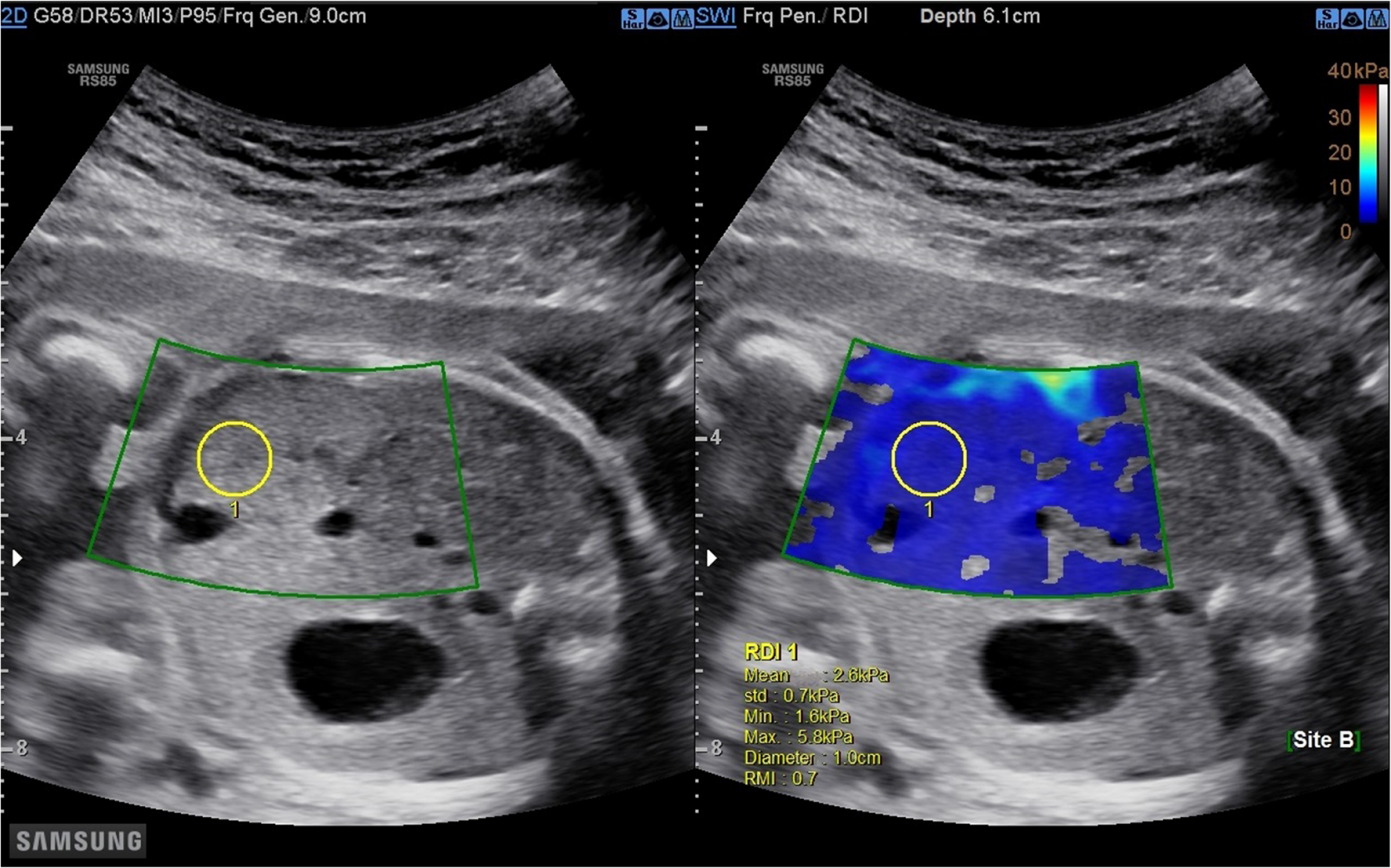
Elasticity measurement of the foetal liver in the region inside the hepatic portal sinus (segment V) with 2D-SWE after obtaining a B-mode image of abdominal circumference

The primary judgement criterion was the value of YM (elasticity modulus) expressed in kilopascals (kPa). A Samsung RS 85 system® with CE certification CE LNE/G-MED (2007/47/EC) equipped with an abdominal convex probe of 1–7 MHz was used for this study. The shear wave acquisition measurement protocol was the same for each patient. Three operators participated in this study. The elasticity of the foetal lung was quantified on the most proximal lung in the region behind the plane passing through the atria after obtaining a B-mode image of a four-chamber view of the foetal heart (Fig. 1). The proximal lung is the one located closest to the ultrasound probe. The elasticity of the foetal liver was quantified in the region inside the hepatic portal sinus (segment V) after obtaining a B-mode image of the abdominal circumference (Fig. 2). In real time, the elasticity appeared colour coded (colour scaled, ranging from 0 to 40 kPa), and the YM value (minimum, maximum, average and standard deviation) at any location was sampled using a round region of interest (ROI) of 10 mm. The distance between the probe and the target organ was recorded for each measurement. Samsung’s S-Shearwave Imaging™ provides an additional reliable measurement index (RMI) determining whether the elasticity value has been correctly measured. This index is calculated by analysing how much the measured shear wave deviates from the theoretical behaviour. An RMI of 0.0 indicates a significant error, whereas an RMI of 1.0 indicates no error [20]. In this study, measurements of YM were considered reliable for an RMI > 0.4. Currently, there is no consensus concerning the number of measurements required to measure elasticity in a tissue explored by 2D-SWE. Studies reporting the use of elastography in newborns, children or adults report a number of measurements varying between 3, 5 and 10 measurements [21]. For the first ten patients included in the study, we calculated the coefficient of variation with the Kruskal-Wallis test for 3, 5 and 10 measurements of YM of the foetal lung and liver. After analysis, there was no significant variation in the average elasticity according to 3, 5 and 10 measurements. In this study, we carried out three successive acquisitions.

### Postnatal variables

The following data were collected: term of birth, weight, neonatal transfer, Apgar score and evaluation of respiratory distress by the Silverman score. In the 2 days after birth, automated evaluation of auditory-evoked potentials (AEPs) was recorded in cases of premature delivery before 37 WG despite cochlear damage in the exposed foetus. After 37 WG, AEPs were performed if otoacoustic emissions were absent.

### Statistical analysis

Trajectories representing the evolution of the foetal lung and liver maturation from at least 3 measurements over time were modelled (24, 28, 32, 36 and 39 WG). The calculation of the effect was carried out from preliminary data obtained in the ELASTOMAP study in a cohort of patients not at risk of preterm delivery [14]. The assumptions underlying the calculation were that a relatively precise estimate (± 5% around the observed values) of the average trajectory with its 95% confidence interval is desired to identify atypical trajectories. For this, we formulated the following assumptions at each time point: an extent of the confidence interval of the mean of 10% of the value of the mean, a dispersion equal to the standard deviation of the mean and an alpha risk of 5%. It was determined that 72 patients should be included in the current study.

The reliability of the measurements was tested according to depth (distance between the probe and the ROI). If the variability of the measurements (coefficient of variation calculated on repeated measurements) did not increase with the depth of the measurements, we deduced that these two parameters were independent in our data. The results were expressed as the mean difference with a 95% confidence interval, standard deviation of the differences and *p* value. A mixed analysis of variance was performed to test lung and liver elasticity values and dispersions at each time point. Pearson’s correlations were used to test the linear link between the depth and the reliability of the measurement.

Statistical analysis was performed with SAS/STAT version 9.4 for Windows.

## Results

During the study period, 77 women with an uncomplicated pregnancy were enrolled prospectively. At least 3 ultrasounds with 2D-SWE were available for 72 women giving birth at term to healthy children without RDS (Silverman score < 4). Five patients were excluded from the analysis: 4 women received corticosteroids for threatened preterm labour, and 1 woman developed gestational diabetes. Maternal characteristics are summarized in Table 1.

**Table 1:** Maternal and infant characteristics

Maternal characteristics	*n* = 72
Maternal age (years)	26 ± 5.26 [19–39]
BMI^*a*^ (kg/m^2^)	23.7 ± 6.14 [19.43–27]
Infant characteristics in the 2 days following birth (lost to follow-up: 2)	*n* = 70
Gestational age at birth	39.6 ± 1.74 [35–40]
Birth weight	3314.86 ± 520.45 [1890–4850]
Median Apgar 1 min	10 ± 1.17 [4–10]
Median Apgar 5 min	10 ± 0.66 [5–10]
Spontaneous ventilation	66 (66/70; 94.2%)
Mechanical ventilation	4 (4/70; 5.8%)
Mechanical ventilation with intubation	0
Normal otoacoustic emissions	64 (64/70; 91.4%)
Normal auditory evoked potentials	2 (2/2; 100%)

^*a*^*BMI*, body mass index

Results are presented as follows: mean ± standard deviation [minimum;maximum]; frequency (X/Y; percentage); *p* < 0.05 is significant (Student’s t test or Mann-Whitney’s U test for quantitative variable; chi-squared test or Fisher’s exact test for qualitative variables, as appropriate)

**Fig. 3:**
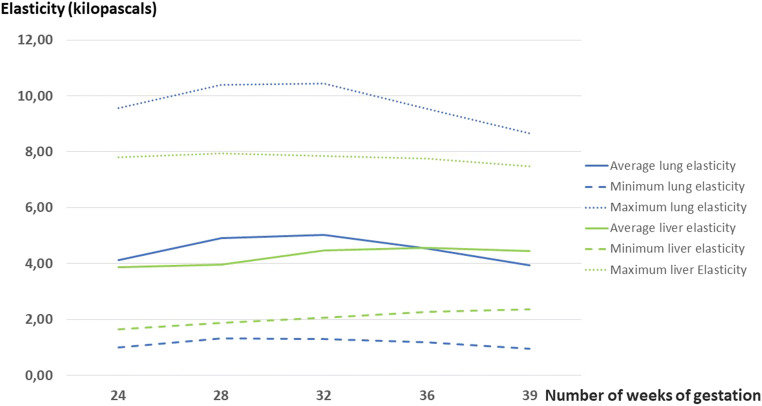
Average, maximum and minimum elasticity values for foetal lung and liver according to gestational age

**Fig. 4:**
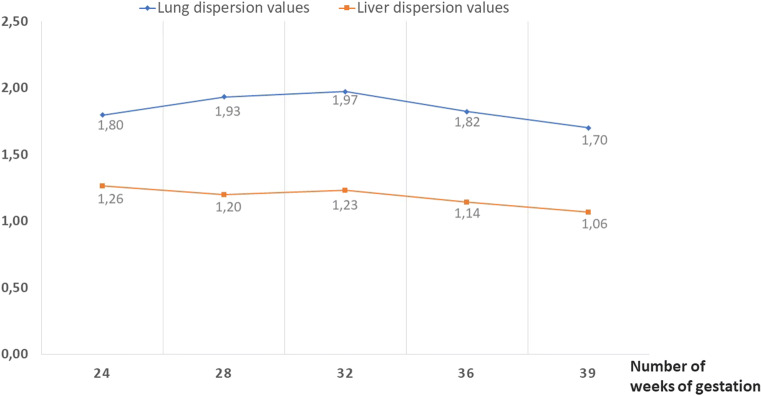
Dispersion of the average elasticity values for foetal lung and liver according to gestational age

The average, maximum and minimum elasticity values for foetal lung and liver according to gestational age are presented in Fig. 3. The evolution of the dispersion of these values according to gestational age is presented in Fig. 4. A mixed analysis of variance showed that the average elasticity for the lung and liver was significantly different from 24 WG to 36 WG (*p* < 0.01). While foetal liver elasticity increased (3.86 kPa at 24 WG versus 4.45 kPa at 39 WG), lung elasticity evolved at different levels. From 24 WG to 32 WG, there was a tendency for lung elasticity to gradually increase (4.12 kPa at 24 WG, 4.91 kPa at 28 WG, 5.03 kPa at 32 WG). This difference was significant (*p* < 0.002). After 32 WG, the lung elasticity decreased to 4.54 kPa at 36 WG and then 3.94 kPa at 39 WG.

The dispersion of the average elasticity values was also different between the organs with a mixed analysis of variance: dispersion was greater for the lung than for the liver between 24 and 36 WG (*p* < 0.0001). For the lung, this dispersion seemed to decrease after 32 WG, while it remained stable for the liver. However, the evolution between 24 and 36 WG was not significant regardless of the explored organ (*p* = 0.40).

The mean distances between the ultrasound probe and ROI placement on the lung or liver remained stable between 24 and 39 WG: 4.6 cm (2.5–8) and 5.8 cm (3–10), respectively. Depending on the depth quartile, there was a moderate variation in the elasticity values. This variation was less important for the liver than for the lung (Tables 2 and 3). For the lung, the Pearson correlation coefficient between the depth of the measurements and the coefficient of variation was 0.19, which was a significant difference (*p* = 0.0008, *n* = 314 measurements). The greater the depth increased, the greater the variability of the measurements increased. However, the increase remained small. In addition, the values were considered valid and therefore repeatable except for the 4th quartile of a depth corresponding to a distance between the probe and the ROI of 8 cm. For the liver, this same Pearson correlation coefficient was 0.08, which was a nonsignificant difference (*p* = 0.15, *n* = 311 measurements). The variability in the measurements cannot be explained by depth. Unlike the lung, the measurements had a coefficient of variation < 0.3 and were considered repeatable regardless of the depth.

**Table 2:** Lung elasticity values according to the depth quartile

	Depth	Average coefficient of variation (± standard deviation)
1^st^ quartile	3.90	0.26 (± 0.15)
2^nd^ quartile	4.50	0.25 (± 0.12)
3^rd^ quartile	5.30	0.29 (± 0.14)
4^th^ quartile	8.10	0.33 (± 0.15)

**Table 3:** Liver elasticity values according to the depth quartile

	Depth	Average coefficient of variation (± standard deviation)
1^st^ quartile	4.60	0.19 (± 0.13)
2^nd^ quartile	5.60	0.20 (± 0.13)
3^rd^ quartile	6.80	0.20 (± 0.11)
4^th^ quartile	10.00	0.23 (± 0.13)

Paediatricians examined a total of 70 children exposed to 2D-SWE in the 2 days following birth (lost to follow-up *n* = 2). All the infants exposed to 2D-SWE were born alive and in good health. Infant characteristics are summarized in Table 1. All the infants were born at 39.6 (+/- 1.7) WG, and otoacoustic emissions were present for 64 infants. AEPs were controlled in 2 infants and were normal. Otoacoustic emissions were not registered in the medical files for 4 infants.

## Discussion

The mean elasticity value and its dispersion evolved differently during gestation between foetal lungs and the liver and could be new prenatal parameters for exploring foetal lung maturity. The simultaneous interpretation of the measurement dispersion and elasticity value is interesting because it could reflect histological modifications of both tissues during gestation. For foetal lungs, the development of stiff structures such as blood vessels and bronchi during the canalicular (16–26 WG) and saccular stages (24 WG-term) precedes that of the soft pulmonary parenchyma, especially before 32 WG. During the exploration of the foetal lung with 2D-SWE, the distribution of vasculo-bronchial elements and parenchyma in the same ROI could influence the elasticity measurement. Before 32 WG, the probability of measuring the stiffness of both vasculo-bronchial elements and foetal pulmonary parenchyma in an ROI is important, leading to a greater dispersion of the elasticity values with a tendency to obtain higher values (4.12 kPa at 24 WG, 4.91 kPa at 28 WG, 5.03 kPa at 32 WG, *p* = 0.002). After 32 WG, the probability of exploring only the lung parenchyma within an ROI appears to be greater, and two phenomena could explain the decrease in foetal lung elasticity and dispersion attenuation (4.54 kPa at 36 WG and 3.94 kPa at 39 WG): development of the pulmonary parenchyma and the production of surfactant. Phosphatidylcholine is one of the most important components of surfactants, and synthesis of this viscoelastic substance can lead to dispersion of SWS and attenuation. As dispersion increases, shear waves will be attenuated [22–24]. For the liver, elasticity seems to increase during gestation (3.86 kPa at 24 WG versus 4.45 kPa at 39 WG), and measurements of dispersion seem constant and may reflect a certain homogeneity of the tissue during gestation.

These results are consistent with the findings of Da Silva et al in ovine foetuses, where SWS in the liver gradually increased during gestation [25]. The use of 2D-SWE in foetal medicine is actually not recommended in current practice, and no health agency has published recommendations about the widespread use of 2D-SWE in obstetrics due to a lack of data concerning the impact of acoustic radiation force on particular displacement in foetal tissues [26]. Although no apparent histologic changes after ultrasound elastography have been reported, the absence of all bioeffects cannot be excluded [27]. Issaoui et al measured heating variations during B-mode, pulse Doppler and 2D-SWE ultrasound imaging with a specific instrumental phantom [28]. The temperature rise caused by SWE was the highest, approximately 2.5 times greater than that caused by pulse Doppler and more than four times greater than that caused by B-mode. The continuous movement of the transducer, freezing of the output and reviewing acquired data during the examination, blood perfusion and the presence of amniotic fluid could reduce the temperature changes deeper in the tissue [28]. Currently, the uncertainty of the bioeffects of acoustic radiation force elastography leads to the recommendation to avoid foetal exposure before 12 WG to ensure that organogenesis is sufficiently completed [29]. The foetal cochlea may be a particularly sensitive structure, as 2D-SWE provides mechanical pulsatile vibration [30]. In our preliminary study, neonatal hypoacusis screening tests were normal for 100 infants exposed to 2D-SWE during the prenatal period. In one case, otoacoustic emissions were not registered in the medical file, and one infant presented abnormal AEP for one ear in one case of premature delivery at 31 WG [14]. With this complementary study, we confirmed that no adverse neonatal outcomes were observed in 70 new infants exposed prenatally to 2D-SWE, and 94.2% (66/70) of them had a negative result in the audiologic tests.

The main limitations of this study include the impact of depth on measurement accuracy and the postulate that the explored tissue is homogeneous. It is accepted that the SWS is underestimated with the depth of the explored organ, but this effect would be limited when exploring soft tissues compared to stiff tissues. It can be explained by a greater damping of the sound pressure in stiff tissues and by a more limited compressibility of the soft tissues [31]. Moreover, estimation of YM values assumes homogeneity of the sample. However, most biological materials, including lung parenchyma, are increasingly heterogeneous during gestation. This heterogeneity can result in artefacts such as variation in YM depending on depth. In our study, there was a moderate variation in the elasticity value depending on the depth. This variation was greater for the lung, and the threshold for the depth that could influence elasticity measurements was a distance between the probe and the ROI of 8 cm. These data are consistent with those in the literature [32–34]. Below this threshold, the intensity of the ultrasonic beam becomes too weak to generate a force of ultrasonic radiation [35]. Another limitation is the non-differentiation between the left and right lungs. Measurements were performed on the proximal lung, which can be the right or the left one depending on the position of the fetus. In this preliminary study, we chose to limit the impact of depth by favouring exploration of the lung closest to the probe, whatever the side. Lung density is lower for the left one, which could have an impact on the measurement of elasticity.

In conclusion, this is the first study to provide normal foetal lung and liver elasticity values from 24 to 39 WG using 2D-SWE in a cohort of healthy newborns without RDS. The simultaneous interpretation of elasticity values and their dispersion is relevant for interpreting stiffness variations in both organs. In the current obstetrical practice, the impact of depth on elasticity measurements with 2D-SWE seems to be limited to a distance between the probe and the target organ under 8 cm. These preliminary results support further research to assess the performance of 2D-SWE to predict foetal lung maturity in clinical practice and to compare elasticity values of foetal lungs in healthy new-borns and those with RDS.

## Abbreviations

2D-SWE: Two-dimensional shear wave elastography
AEP: Auditory evoked potentials
BMI: Body mass index
IUGR: Intra-uterine growth restriction
kPa: Kilopascals
MHz: Megahertz
RDS: Respiratory distress syndrome
RMI: Reliable measurement index
ROI: Region of interest
SWE: Shear wave elastography
SWS: Shear wave speed
WG: Weeks of gestation
YM: Young’s modulus

## Declarations

### Guarantor

The scientific guarantor of this publication is Dr Nicolas Mottet.

### Statistics and biometry

Two of the authors have significant statistical expertise (Lionel Pazart and Chrystelle Vidal).

### Methodology

• prospective

• observational

• multicenter study

## References

[1] Balestrini JL, Niklason LE (2015). Extracellular matrix as a driver for lung regeneration. Ann Biomed Eng.

[2] Suki B, Stamenovic D, Hubmayr R (2011). Lung Parenchymal Mechanics. Compr Physiol.

[3] Torday JS, Sanchez-Esteban J, Rubin LP (1998). Paracrine mediators of mechanotransduction in lung development. Am J Med Sci.

[4] Fung YC (1975). Does the surface tension make the lung inherently unstable?. Circ Res.

[5] Gennisson J-L, Deffieux T, Fink M, Tanter M (2013). Ultrasound elastography: principles and techniques. Diagn Interv Imaging.

[6] Bercoff J, Tanter M, Fink M (2004). Supersonic shear imaging: a new technique for soft tissue elasticity mapping. IEEE Trans Ultrason Ferroelectr Freq Control.

[7] Carlson LC, Feltovich H, Palmeri ML, Dahl JJ, Munoz del Rio A, Hall TJ (2014). Estimation of shear wave speed in the human uterine cervix. Ultrasound Obstet Gynecol.

[8] Alison M, Biran V, Tanase A (2015). Quantitative shear-wave elastography of the liver in preterm neonates with intra-uterine growth restriction. PLoS One.

[9] Abeysekera JM, Ma M, Pesteie M (2017). SWAVE imaging of placental elasticity and viscosity: proof of concept. Ultrasound Med Biol.

[10] Alici Davutoglu E, Ariöz Habibi H, Ozel A, Yuksel MA, Adaletli I, Madazlı R (2017). The role of shear wave elastography in the assessment of placenta previa-accreta. J Matern Fetal Neonatal Med.

[11] Arioz Habibi H, Alici Davutoglu E, Kandemirli SG (2017). In vivo assessment of placental elasticity in intrauterine growth restriction by shear-wave elastography. Eur J Radiol.

[12] Quarello E, Lacoste R, Mancini J, Melot-Dusseau S, Gorincour G (2016). ShearWave elastography of fetal lungs in pregnant baboons. Diagn Interv Imaging.

[13] Quarello E, Lacoste R, Mancini J, Melot-Dusseau S, Gorincour G (2015). Feasibility and reproducibility of ShearWave(TM) elastography of fetal baboon organs. Prenat Diagn.

[14] Mottet N, Cochet C, Vidal C (2020). Feasibility of two-dimensional ultrasound shear wave elastography of human fetal lungs and liver: a pilot study. Diagn Interv Imaging.

[15] Beck APA, Araujo Júnior E, Leslie ATFS, Camano L, Moron AF (2015). Assessment of fetal lung maturity by ultrasound: objective study using gray-scale histogram. J Matern Fetal Neonatal Med.

[16] Gorincour G, Bach-Segura P, Ferry-Juquin M (2009). Lung signal on fetal MRI: normal values and usefulness for congenital diaphragmatic hernia. J Radiol.

[17] Moshiri M, Mannelli L, Richardson ML, Bhargava P, Dubinsky TJ (2013). Fetal lung maturity assessment with MRI fetal lung-to-liver signal-intensity ratio. AJR Am J Roentgenol.

[18] Palacio M, Bonet-Carne E, Cobo T (2017). Prediction of neonatal respiratory morbidity by quantitative ultrasound lung texture analysis: a multicenter study. Am J Obstet Gynecol.

[19] Donda K, Vijayakanthi N, Dapaah-Siakwan F, Bhatt P, Rastogi D, Rastogi S (2019). Trends in epidemiology and outcomes of respiratory distress syndrome in the United States. Pediatr Pulmonol.

[20] Foncea CG, Popescu A, Lupusoru R (2020). Comparative study between pSWE and 2D-SWE techniques integrated in the same ultrasound machine, with Transient Elastography as the reference method. Med Ultrason.

[21] Kim DW, Suh CH, Kim KW, Pyo J, Park C, Jung SC (2019). Technical performance of two-dimensional shear wave elastography for measuring liver stiffness: a systematic review and meta-analysis. Korean J Radiol.

[22] Carlsen JF, Pedersen MR, Ewertsen C (2015). A comparative study of strain and shear-wave elastography in an elasticity phantom. AJR Am J Roentgenol.

[23] Carstensen EL, Parker KJ, Lerner RM (2008). Elastography in the management of liver disease. Ultrasound Med Biol.

[24] Barry CT, Mills B, Hah Z (2012). Shear wave dispersion measures liver steatosis. Ultrasound Med Biol.

[25] Silva PDA, Uscategui RAR, Santos VJC (2019). Acoustic radiation force impulse (ARFI) elastography to asses maternal and foetal structures in pregnant ewes. Reprod Domest Anim.

[26] Issaoui M, Debost-Legrand A, Skerl K (2018). Shear wave elastography safety in fetus: A quantitative health risk assessment. Diagn Interv Imaging.

[27] Sugitani M, Fujita Y, Yumoto Y (2013). A new method for measurement of placental elasticity: acoustic radiation force impulse imaging. Placenta.

[28] Issaoui M, Miloro P, Balandraud X (2020). Temperature elevation in an instrumented phantom insonated by B-mode imaging, pulse Doppler and shear wave elastography. Ultrasound Med Biol.

[29] Massó P, Melchor J, Rus G, Molina FS (2020). A preliminary study on the safety of elastography during pregnancy: Hypoacusia, anthropometry, and Apgar score in newborns. Diagnostics (Basel) 18.

[30] Massó P, Rus G, Molina F (2017). On the safety of elastography in fetal medicine: a preliminary study of hypoacusia. Ultrasound Obstet Gynecol.

[31] Aubry S, Risson J-R, Kastler A (2013). Biomechanical properties of the calcaneal tendon in vivo assessed by transient shear wave elastography. Skelet Radiol.

[32] Shin HJ, Kim M-J, Kim HY, Roh YH, Lee M-J (2016). Comparison of shear wave velocities on ultrasound elastography between different machines, transducers, and acquisition depths: A phantom study. Eur Radiol.

[33] Zhao H, Song P, Urban MW (2011). Bias observed in time-of-flight shear wave speed measurements using radiation force of a focused ultrasound beam. Ultrasound Med Biol.

[34] Dhyani M, Xiang F, Li Q (2018). Ultrasound shear wave elastography: Variations of liver fibrosis assessment as a function of depth, force and distance from central axis of the transducer with a comparison of different systems. Ultrasound Med Biol.

[35] Bouchet P, Gennisson J-L, Podda A, Alilet M, Carrié M, Aubry S (2020). Artifacts and technical restrictions in 2D shear wave elastography. Ultraschall Med.

